# Understanding the perspective of women who use the Billings Ovulation Method®: a focus group study

**DOI:** 10.1186/s12905-023-02398-w

**Published:** 2023-05-10

**Authors:** Montserrat Ayala-Ramirez, Mary E. Grewe, Julie Kaiser, Emily Kennedy, Martha Winn, Rachel Peragallo Urrutia

**Affiliations:** 1 Billings Ovulation Method Association, St. Cloud, MN United States of America; 2 Present Address: Office of Outreach and Health Disparities. One Baylor Plaza, Suite 450A, Houston, TX 77030-3411 United States of America; 3 North Carolina Translational and Clinical Sciences (NC TraCS) Institute, University of North Carolina, Chapel Hill, NC United States of America; 4 Reply Fertility, PLLC, Cary, NC United States of America; 5 Department of Obstetrics and Gynecology, The University of North Carolina, Chapel Hill, NC United States of America

**Keywords:** Billings ovulation method, Fertility awareness-based method, Fertility control, Natural fertility, Natural family planning methods

## Abstract

**Background:**

The Billings Ovulation Method®(the Billings Method) is a fertility awareness-based method (FABM) of family planning that relies on the observation of patterns of fertility and infertility based on vulvar sensations and appearance of discharges. This allows people to choose when to have intercourse, depending on whether they want to avoid or achieve pregnancy. Few studies have documented user experiences with FABMs.

**Methods:**

We conducted four virtual focus groups (FGs) in May and June 2021 with current adult women users of the Billings Method. We asked questions about users' reasons for selecting a FABM and the Billings Method, positive experiences and challenges learning and using the Billings Method, and suggestions for improving the user experience. We performed a content analysis of the transcribed FGs to explore key themes from the discussions. COREQ guidelines were followed.

**Results:**

Twenty women between the ages of 23 and 43 participated in the FGs. Reasons women described choosing a FABM included to follow religious beliefs, to avoid side effects of hormonal contraception, and/or to learn more about their bodies. Reasons for selecting the Billings Method included perceiving it as more precise and easier to understand than other FABMs, having a scientific basis, and being recommended by family and friends. Experiences related to learning and using the Billings Method were mainly positive. They included finding the method easy to use and learn, successfully using it to either postpone or achieve a pregnancy and increasing their awareness of their bodies. Challenges for participants included the inherent learning curve for identifying sensations at the vulva and the required periods of abstinence. Participants provided suggestions and recommendations for improving users' experience, including raising awareness of the Billings Method among healthcare providers.

**Conclusions:**

Users of the Billings Method expressed an overall positive experience when learning and using it for family planning and body awareness. Some challenges were identified that offer opportunities to improve how the Billings Method is taught and delivered. These findings can also enhance healthcare providers' interactions with FABM users, including those of the Billings Method.

**Supplementary Information:**

The online version contains supplementary material available at 10.1186/s12905-023-02398-w.

## Background

Fertility Awareness-Based Methods (FABMs) are a group of family planning methods with precise protocols to teach people how to track changes in one or more biomarkers to identify more or less fertile days during each menstrual cycle. Users can then determine when and whether to have intercourse based on their desires to plan or avoid pregnancy [1, 2]. FABMs require partner communication and collaboration and can be taught by trained community members. Natural Family Planning (NFP) is a related term to describe a particular way of using FABMs in a spiritual or religious setting which typically involves periodic abstinence and not combining it with the use of any other contraceptive method (e.g., barrier methods) [3].

An advantage of FABMs is that they avoid using hormones or devices that may cause side effects or religious concerns. In addition, they may help the user better understand their own body, monitor health, and avoid or achieve a pregnancy [4]. FABMs are compatible with the teachings of major world religions, unlike other family planning methods [5, 6]. On the other hand, some disadvantages include the periods of abstinence required, the lower effectiveness compared to other contraceptive methods, and the heavy reliance on the correct use from the user [7].

The Billings Ovulation Method® (the Billings Method) is a FABM that can be used by people with a variety of reproductive histories, including people with long and irregular cycles, those who are lactating, amenorrheic, and or perimenopausal [1, 8–10]. Billings Method instruction is available in many countries and languages and has been used with visually impaired [11] and low literacy populations [12]. It relies on tracking patterns of fertility and infertility in the woman by observations of vulvar sensations and observance of any discharges which may be indicative of cervical response to ovarian activity [13]. Users of the Billings Method track sensation at the vulva and the appearance of any discharge noticed as they go about their normal daily activities. Users learn to be aware of their fertile and infertile patterns and cycle phases. If they decide to avoid pregnancy, they will then apply four rules to understand when intercourse is less likely to lead to pregnancy (Table 1) [14]. For users of the Billings Method, the first-year probability of unplanned pregnancy is 1.1–3.4% with perfect use (correct and consistent use) and ranges from 10–33.6% for typical use [1].

**Table 1:** Four rules to avoid pregnancy in the Billings Method [14]

Early Day Rule 1	Avoid intercourse on days of heavy bleeding during menstruation
Early Day Rule 2	Alternate evenings are available for intercourse during the BIP^a^
Early Day Rule 3	Any change in the Basic Infertile Pattern in sensation or appearance (including bleeding) wait. If the Peak is identified, apply the Peak Rule. If the Peak is not identified and the BIP returns, wait for three full consecutive days of the BIP, and then intercourse is available on the 4th evening of the BIP. Then Early Day Rule #2 applies
Peak^b^ Rule	Intercourse is available every day at any time from the morning of the 4^th^ day after Peak until the end of the cycle

^a^*BIP* Basic infertile pattern is defined as an unchanging pattern in the preovulatory phase. The BIP can be an unchanging pattern of dry (nothing sensed at the vulva/nothing seen) or an unchanging pattern of discharge

^b^Peak Criteria is a changing and developing pattern, ending in a slippery sensation with an abrupt change to no longer slippery or wet. The Peak Day is the last day of the slippery sensation in this pattern

Despite the scientific evidence demonstrating that the Billings Method can be used to avoid pregnancy, there is still a lack of information and many misconceptions about Billings and other FABMs within the lay and medical communities [7, 15]. The Billings Method is used by a small proportion of reproductive-aged women in the United States; however, two analyses of the National Survey of Family Growth showed the use of FABMs, in general, increased from 1.1% to 2.2% between 2008 and 2014 [16] and from 2.9 to 3.4% between 2013–2017 [17]. Little is known about why people choose FABMs, the Billings Method, or their user experience. Thus, we designed a qualitative study using focus groups (FGs) to elicit the perspectives of Billings Method female users to understand better why they chose it and their experiences when learning and using it.

## Methods

### Study design

We conducted a qualitative research study using a qualitative description approach [18] to better understand the Billings Method users' beliefs, attitudes, experiences, perceptions, and behaviors.

### Recruitment

We recruited mainly a convenience sample of adult women who had completed training in the Billings Method with a certified instructor or teacher in practicum from the Billings Ovulation Method Association – USA (BOMA-USA). We identified potential participants using registries from certified instructors or teachers in the practicum of BOMA-USA. We invited participants via email or phone calls. We also asked BOMA-USA to send study information to their email distribution list and post it on their Facebook page. In addition, in an effort to diversify and balance our sample to include non-religious perspectives and increase participation of underrepresented demographics and various religions, we asked several Billings Method instructors to share study information with their trainees and clients with these characteristics. Interested participants were asked to email a research team member (MAR) to express interest in the study. They then received an email to schedule an enrollment call. We included those who completed their training 48 months prior to enrollment and identified the Billings Method as their primary method of family planning at the time of enrollment. We excluded women using hormonal contraceptives or contraceptive implants and those who were pregnant or in menopause. During the enrollment call, a research team member (MAR) confirmed eligibility, scheduled the participant in an FG session, introduced the principal investigator, the research staff, research goals, and interests, conducted and documented verbal informed consent. Members of the research team who were Billings Ovulation Method instructors (MAR, JK, MW, EK) did not participate in any sessions that included participants they had instructed. All the methods in this study were performed in accordance with the relevant guidelines and regulations found in the 2013 Declaration of Helsinki [19]. The University of North Carolina Institutional Review Board at Chapel Hill approved the study including the “Verbal Consent Script for Telephone Call,” which was read and consented to by each of the study participants (IRB #19–1966). After the FG sessions, each participant received a virtual $30 gift card to thank them for participating, regardless of how long they remained in the FG discussion.

### Data collection

We conducted four virtual FGs of ~ 90 min each via Zoom between May and June 2021. An experienced female facilitator who had no prior relationship to the participants (MG) led a semi-structured discussion using a focus group guide. After the first session, we made moderate changes to the guide based on the participants’ understanding of the questions to enhance clarity (Table 2). The facilitator started each session by introducing herself, the research staff, and research goals, followed by facilitating a conversation on topics including reasons for selecting a FABM in general and the Billings Method specifically, positive experiences and negative experiences/challenges when learning and using the Billings Method, and suggestions for improving the experience of women using the Billings Method. The FGs sessions were audio and video recorded. Recordings were saved in a password-protected institutional server. During each session, at least one research team member took field notes for future reference. Participants were also asked to complete a demographic survey via Qualtrics.

**Table 2:** Focus group discussion guide (final version)

1. What were the reasons you chose to use a Fertility Awareness Based or a Natural Family Planning Method? a. What were the reasons you decided to use the Billings Ovulation Method specifically? Probes: i) How did you learn about the Method? ii) What were the reasons you chose the Method over other methods you were considering?
2. Next, we’d like to learn more about your experiences using the Billings Ovulation Method, including both positive and negative experiences. What has been your experience using the Billings Ovulation Method? Probes: a. What has been your experience learning the Method? b. What has been your experience tracking your cycle? c. What has been your experience planning a pregnancy?
3. What challenges have you faced using the Billings Ovulation Method?
4. [SKIP IF SHORT ON TIME] What suggestions do you have for improving the experience of women using the Method? Probe: a. How would you change the way the Method was delivered or the learning materials? b. How can challenges to using the Method be addressed?
5. How has using the Billings Ovulation Method affected you and your life, in positive or negative ways? Probes (if not discussed previously): a. How has using the Billings Ovulation Method affected your physical health? Your emotional and mental health? b. How has using the Billings Ovulation Method affected your relationship with your partner or partners? c. How has your understanding of your body and your fertility changed since using the Billings Ovulation Method?
6. Closing question: Before we end our discussion, is there anything else you would like to share with us about your experience using the Billings Ovulation Method?

### Data analysis

Members of the research team transcribed the recordings (MAR, JK), then de-identified and reviewed the transcripts for quality (JK, MG). Transcripts were not returned to participants for review. FG transcripts were then content analyzed to understand key areas of interest and to identify emerging themes. An initial codebook was developed based on the FG discussion guide (e.g., “challenges,” “suggestions”). Two members of the team (MAR, MG) independently coded the first transcript, adding additional emerging codes as needed (e.g., “Perception of contraceptives,” “partner perception/involvement”) (See Additional File 1 for the description of the final version of the codebook). Transcripts were reviewed, discrepancies were discussed until a consensus was reached, and the codebook was updated. This process was repeated for all four transcripts. As there were significant changes to the codebook after transcripts 1 and 2, the first two transcripts were reviewed a second time to ensure coding consistency.

Atlas.ti v.8 [20] was used to manage the coding process (MG), and transcripts were updated in Atlas.ti with final codes after consensus coding discussions. Query functions were utilized to categorize data further (e.g., “Using Billings to monitor health/body OR “using Billings for family planning” AND “negative experiences/challenges”). Finally, code reports were developed and reviewed to explore and describe themes. During the data analysis process, we confirmed that we had reached data saturation and that no additional focus group discussions were required, even after specifically sampling for different religious perspectives in the last group. Participants were not requested to provide feedback on the findings.

### Quality and trustworthiness

In order to ensure the quality and trustworthiness of the study, the following strategies were employed: prolonged engagement (four 90-min focus group discussions), triangulation (multiple researchers engaged in data analysis), providing a thick description of results, providing a description of the sampling strategy, discussing findings in the context of the literature, assessing for data saturation, utilizing an iterative data analysis process, and keeping an audit trail of the coding process [21]. In addition, we completed the COnsolidated criteria for REporting Qualitative research (COREQ) checklist (See Additional File 2_COREQ).

## Results

### Participants' characteristics

A total of 20 women participated in four FG discussions of five participants each. One person withdrew after enrollment due to an unforeseen family matter. Characteristics of the participants are presented in Table 3. The median age of participants was 32; half identified as White, most were married and had experienced pregnancy previously, and more than half identified as Catholic.

**Table 3:** Focus group demographic characteristics

Participants Characteristics	*n* = 20 (%)
**Age (years)**	
Min	23
Max	43
Median	32
**Marital Status**	
Married	15 (79)
Single	2 (10.5)
Living with partner	1 (5.3)
Other	1 (5.3)
Missing	1 (5)
**Race**^a^	
Asian	1 (5.3)
Black / African American	1 (5.3)
Other	7 (37)
White	10 (53)
Missing	1 (5)
**Language spoken at home**	
English	17 (89.5)
Spanish	2 (10.5)
Missing	1 (5)
**Number of pregnancies**	
None	1 (7.7)
1–2	6 (46.1)
3–4	3 (23)
5–6	3 (23)
Missing	7 (35)
**Highest level of education**	
Graduate/Professional degree	7 (37)
4-year degree	9 (47)
2-year degree	1 (5.3)
Some college	1 (5.3)
High School Graduate	1 (5.3)
Less than High School	0
Missing	1 (5)
**Religion**	
Christian Catholic	11 (61)
Christian Other	3 (17)
Unaffiliated	4 (22)
Missing	2 (10)
**Years using the Billings Method**	
Less than one year	6 (31.6)
One year	2 (10.5)
Two years	6 (31.6)
Three years or more	5 (26.3)
Missing	1 (5)
**Ways of Charting**	
Online	9 (47.4)
Paper	4 (21)
Don't chart	1 (5.3)
Other	5 (26.3)
Missing	1 (5.3)
**Certified Instructor**	
Certified	12 (66.7)
On-Practicum	6 (33.3)
Missing	2 (10)
**Finished Instruction**	
Online	10 (55.5)
In-Person	6 (33.3)
Online / In-Person	2 (11.1)
Missing	2 (10)

^a^Six "Other" identified as Latina or Hispanic in origin

We focused our qualitative analysis on the following areas: reasons for choosing a FABM, reasons for selecting the Billings Method specifically, positive and negative experiences while learning and using the Billings Method, and suggestions for improving the delivery of the Billings Method (Table 4).

**Table 4:** Themes and subthemes

Category	Examples
Reasons for choosing FABM	- Religious beliefs discouraging the use of contraceptives - Wanting a "natural" method - Concerns about side effects of hormonal birth control - Desire to learn more about their bodies
Reasons for selecting the Billings Method specifically	- Easy to understand and simple to use - Affordable - Backed by science and effectiveness - "Word of mouth."
Learning the Billings Method—Positive experiences	- A new way to think about themself - Feels more "natural." - No need to do internal investigations^a^ or take a daily basal body temperature - Having an experienced and patient instructor
Using the Billings Method- Positive experiences	- Achieving pregnancy is easy - To postpone a pregnancy, one needs to use the four rules of the Billings Method correctly - Partner support
Learning and using the Billings Method- Challenges and negative experiences	- Learning curve - Charting every day on paper - Getting pregnant - More religious messaging than necessary; instructors teaching using their religious perspective - Charting with PCOS and during breastfeeding

^a^Internal investigations refer to introducing the fingers in the vagina to touch and feel the mucus

### Reasons for selecting a FABM in general

Many participants described utilizing FABMs for religious reasons, and in some FGs, nearly all participants described this as their primary motivation for using a FABM. Many decided to use a FABM to follow Catholic teachings, with some referencing what was taught in marriage preparation classes within their church.

“My primary reason [and] for my husband… is that I am Catholic, so...whatever family spacing we decided to do, we wanted to do so in accordance with the Catholic Teachings. (FG2)”

Some participants described pursuing a FABM for religious reasons and a general desire to use a "natural" method. This belief appeared to be intertwined for some, feeling that it was essential to keep their body in a natural state for spiritual or religious purposes:

“…It was clear to me that I wanted to learn how to do [family planning] without any chemicals, without anything that would cause me side effects or give me problems to get pregnant in the future, because I knew families are supposed to be fertile and have kids and it was according to the teachings of the Catholic Church. (FG2)”

“When [you] don't alter your menstrual cycle, you are following God's design... God likes order... for example, [in] the water cycle. If you alter something,[if] you cut every tree you see or forever [have] no rain, there might be consequences... It's the same for us; when you alter your menstrual cycle, there will be consequences. (FG1)”

Similarly, some participants described discomfort with personally "controlling" their fertility with hormonal birth control.

“As a Christian, I have a conviction not to use anything very permanent as far as birth control goes... I want to give God the opportunity to overrule me should He see fit… (FG3)”

“I did not want to get into birth control and prevent it [conception, pregnancy]. Because again, they [my babies] truly were miracles... I just felt like, who am I to prevent that?...at the same time, I wasn't ready to get pregnant again. (FG1)”

In addition, many participants discussed concerns about the side effects of hormonal birth control and the desire to avoid these, leading them to use FABMs. For example, some women described experiencing negative mood-related symptoms while using hormonal birth control. Others discussed having health issues like Polycystic Ovary Syndrome (PCOS), for which their doctors wanted to prescribe hormonal birth control but were concerned that this would lead to side effects or future fertility issues.

“I first got into fertility awareness because I didn't want to keep using hormonal birth control. I didn't have any severe side effects, but it did affect my mood and stuff, so I was kind of over that. (FG4)”

“I knew the side effects that [hormonal birth control] could cause on your body, and I always knew that I do want kids in the future. I wanted to do everything in my power to allow that to happen… so I just wanted to do[it] the natural way… (FG1)”

Finally, some participants described pursuing a FABM because of general interest and desire to learn more about their bodies or, as one participant noted, to share this information with her children more effectively.

“I felt like something was kind of off, so I decided to research a method that could tell me more about my female health and my hormones. (FG2)”

“I've learned several different [FABMs], ... I'm extremely interested in all methods of NFP; to be honest, I'll probably learn more as I go on in my life just because I'm (a) kind of a learner and (b) I find it really interesting. (FG4)”

### Reasons for selecting the Billings Method

When describing why they chose the Billings Method specifically, as opposed to other FABMs, one of the most common reasons participants cited was that it seemed easy to understand and simple to use. In addition, the Billings Method does not require taking temperatures, tracking multiple indicators, or having access to additional technology like apps or devices.

“Billings[seemed] a lot more simple than the other methods. (FG2)”

“[The Billings Method] is just a few things to remember, and that's it. It's safe, and it's simple. (FG4)”

“…It was so hard to understand … Creighton and Napro-technology things, and there were all these options out there, and I didn't feel I needed all that… so I thought, well, if [The Billings Method is] the simplest one, and it's only about appearance and sensation, then I think that's just fine. (FG2)”

One participant highlighted the affordability of the Billings Method compared to another method requiring purchasing urinary hormone test strips to monitor fertility:

“With Billings, there isn't that continuous monthly investment that I had to make through the strips, so there is the cost-effective aspect. (FG2)”

Similarly, two participants noted that the Billings Method offered an easier or more affordable path to becoming a natural family planning instructor.

“…They told me [for] Billings, you don't need any prerequisites to become an instructor, so that is how I got into Billings. (FG1)”

In addition to ease of use and simplicity, many participants selected the Billings Method because of perceptions of scientific origins and effectiveness.

“When [we] were looking at different methods and reading about different methods, there was a lot of science behind Billings, which as a scientist was attractive to me… the publications in peer-reviewed scientific journals. (FG2)”

“I wouldn't have considered other NFP…[methods] because I didn't feel that they were consistent and safe enough, but when I heard …[that] the Billings Method was based in science, really grounded in that, and then reading through the book, feeling comfortable with that…(FG3)”

Finally, many participants shared that they selected the Billings Method primarily due to recommendations from others or because they were offered an opportunity to learn this method in particular. Participants described learning about the Billings Method through their workplace, church, friends and family, school, and other social network groups (e.g., Fertility Awareness Groups).

“People [were] referring to it [the Billings Method] constantly, and it wasn't like I did a big online research or anything. I just kind of trust the people who I went to school with. (FG2)”

“I chose Billings because it just happened to be the method my coworker was teaching, and she needed students, so she was like, I'll practice with you, and that's how I got into Billings. (FG3)”

“I was seeing a lot of talk about Billings on the online groups. I saw a lot of people I found to be knowledgeable about fertility awareness, in general, promoting it, just talking about how interesting it was and how it's great for people with irregular cycles and things like that, so it piqued my interest… (FG4)”

### Learning and using the Billings Method: positive experiences

Many women expressed that learning and using the Billings Method was clear and easy. They found it easier to understand and required less charting than other methods, and noted that it is descriptive in terms of explaining the anatomical and physiological processes involved.

“[The Billings Method instruction is] descriptive of anatomy, how your cycles work, and exactly how to use the method. On this day, avoid this, track this; it was very like day by day, like it was really… easy to follow and just descriptive, I guess. (FG2)”

“I think Billings is the easiest method because it requires little charting; I guess that's how … I compared [it] to other methods. (FG1)”

Some participants mentioned that having experienced and patient instructors who provided one-on-one meetings and spent time reviewing charts helped them understand the Billings Method and gain confidence in their observations and sensations. Positive instruction approaches included simplifying the terminology, helping with the "overthinking" about their sensations (FG4), and asking questions that were helpful to understanding situations affecting the participants' menstrual cycle (e.g., "what is happening in your life?") (FG1). The support of a knowledgeable instructor was appreciated by two women who were breastfeeding and had more challenges identifying their potential times of fertility.

“I am breastfeeding, so my hormones are on [a] rollercoaster, right? So, she [the instructor] was trying to work with me to see, ok [it] is hard for us to find a pattern; however, we will spend time looking through the chart, we [will] meet more often just because I'm breastfeeding, and now finally, ok, this is your pattern, now you can go on your own. But it was kind of having a coach. (FG4)”

In the context of using the Billings Method, some participants described successfully using it to time intercourse and eventually conceive.

“…We know when they [our daughters] were conceived. Literally, we know when they were conceived. So, it's incredibly effective, from my point of view. (FG2)”

“And I said, "Hey! here it comes an ovulation"…. I was able to take what I learn[ed] then and apply it to my own body and recognize the symptoms of fertility...and I [told] my husband … so… we were able to achieve [a pregnancy]. (FG1)”

Similarly*,* other participants described successfully using the Billings Method to postpone a pregnancy:

“I was able to utilize it kind …[of] for both [planning and postponing a pregnancy], and again it was a matter of when we were ready, we were able to use it to postpone …I remember thinking I might have a medical procedure coming up, I was like, I can't get pregnant before that, so I have to [postpone]. It was wonderful to rely on that to time things and have that peace of mind. (FG1)”

“I think, like using it for contraception, I feel confident … I'm just conservative, and I use the rules, and if I have any blip or change, count to three and then go to the next day in the evening… [The Billings' Method rules are] so powerful. They work, and it's amazing. I think that's why I think Billings is so amazing. You can take all of the science and break it down to Four Rules; if you follow the Four Rules, you're fine and can have control over wherever you want to be. (FG3)”

For most participants, their partner's involvement, support, and participation were appreciated, acknowledged, and influential across the groups. However, partner involvement varied. Types of involvement participants mentioned included their partner being willing to help educate others, their partner supporting them in timing intercourse, and their partner being open to talking about and aware of where they were in their cycle. In addition, some participants noted that their partners helped keep track of their observations and charting.

“I think for our relationship, me and my husband, (sic) it's been positive: one because he was interested in using the method, and [two because] I was interested in the effectiveness of it... [I was] hesitant at first, and [it] ended up being great that we can both be on the same page. I mean, we can communicate well, but just being able to look at the app at the same time is another form of communication in our relationship, which is wonderful. It's been a positive experience for us. (FG2)”

“I think involving my partner hasn't been a big challenge cause he's willing to hear me talk about this stuff. Like I said, I keep my chart on our fridge, so he knows what's happening with it, but he's not much of like the kind of person that will sit in my sessions with me, so he'll hear it more second-hand. He doesn't know the rules by heart, but he's getting there, and it's just making sure that he's involved, and it's not just on me. It's more of a partnership. (FG4)”

Many participants across the four groups expressed appreciation for developing a better understanding of their bodies, including feeling more in tune with themselves and gaining a sense of empowerment through knowledge and awareness.

“The menstrual cycle is so important; it's vital, like our 6th vital sign; if that's off, there's something else that's off. I feel empowered, so I can talk to my healthcare provider and tell her, look, this is what I've observed in my body. (FG3)”

“With symptothermal methods, I wasn't confident in my knowledge of like mucus patterns and how that works in the body, and Billings is a lot more in-depth in that respect, so I appreciated that about the method, and my understanding of that part of it improved, so it's been cool. (FG4)”

### Challenges and negative experiences when learning and using the Billings Method

A challenge shared by many women across the groups was the learning curve for identifying sensations at the vulva, charting, and identifying patterns. Participants described initial difficulty in identifying those sensations and patterns but agreed that it became easier with practice and gaining awareness over time. Some women expressed being confident with their observations after about three to six months, at which time they could more clearly identify patterns in their cycle.

“Once you have more practice, you are able to compare. Once you're able to determine exactly when your fertile period is, you are stable [and] also able to compare your previous month, and then you just become more confident. Moderator: How long do you think it took you to feel confident in that pattern? Participant: Four months. (FG2)”

“…Actually, there is a pattern here of infertility and fertility. It's just like taking that time to understand and being able to learn how to describe what you're feeling. It's a huge learning curve for a lot of people… it's that people are willing to commit to… that learning curve; it's just that I think we need to have more grace with ourselves as new users…(FG2)”

For some participants with PCOS or who were breastfeeding, charting and identifying sensations was especially challenging, and a couple of participants acknowledged that advice from instructors was beneficial to ease the learning process.

“With breastfeeding, it's been really interesting just trying to find the infertile patterns; I feel that's been my biggest challenge lately… The BIP establishment during breastfeeding has been my most difficult. (FG1)”

“…For me, learning it initially was hard, especially because I already knew I had PCOS, but obviously, when I was never dry… but then after time and learning a little bit more, I [am] still trying to get the hang of it I guess… It's better now. I got the hang of it, and after speaking with my instructor and such, she was understanding and helpful. (FG1)”

“We [my instructor and me] started up having meetings again because… I've had constant mucus and not very consistent… not to a degree where I'd be able to establish it as part of my BIP [Basic Infertile Pattern]; so, they reach[ed] out to another instructor out in Florida who was more familiar with someone who has PCOS. So, there again, they're helping me with reevaluating my charts and seeing if I need to adjust how I'm interpreting my sensations and stuff, kind of help[ing] me, which is nice, it's nice to know that there is such a large network (FG2)”

Beyond the normal learning curve while using the Billings Method, daily charting and charting using paper were challenging. Participants expressed that charting on single pieces of paper can be difficult because papers are easily misplaced and difficult to transport, making daily charting burdensome. Some participants overcame this by using Billings Method-approved apps, but not all enjoyed the experience.

“Charting on paper was never a thing that was ever going to work for me long term because I'm mobile, too many things going on. I needed to do something online, and part of why it might have been challenging is that the NFP charting app is a disaster. It's broken, it doesn't always work, it crashes, you can't download things, there are errors, and you have to be focused on refreshing certain things; it's really hard to use. (FG3)”

“I have my notebook because the challenge was single sheets of paper, that was chaotic. How do you compare? Where did you leave it? …you can print out all the paper you want, but it ends [up] being single sheets, right? And so, I ended up creating my notebook, and I love it on paper now cause I can flip and compare but only because I had to struggle with the app at the beginning. (FG2)”

“For me, it was super hard to get to charting every day. Mostly especially cause of it being on paper, it was super hard to do that. (FG2)”

Some users experienced stress and worry while learning or using the Billings Method due to being overwhelmed by the information, inexperienced in using the method, or concerned about getting pregnant*.*

“I get overwhelmed with science. I think it is beautiful and amazing, but when I was in training, that was something overwhelming for me, learning the hormonal changes in the charts, like the actual charts of the different levels of progesterone and all of that, and I was like "wow, this is a lot." (FG1)”

“I cannot get pregnant right now; this would be bad for my family. So, I feel that stress a little bit anyway. (FG3)”

Indeed, two participants shared that they experienced an unintended pregnancy while using the Billings Method. However, both continued using the method after the unexpected pregnancy.

“I mentioned at the beginning that we were not expecting to be pregnant; it is still a wonder which is beautiful. But that I was in that process, my husband and I were overjoyed, and I know that's probably not always the expression that a couple has when they are not expecting to be pregnant. (FG2)”

In one case of unintended pregnancy, the user felt that one cause was improper instruction from the Billings instructor.

“…the last time that I got pregnant while using Billings, my chart said wet, wet, very wet, with sore nipples, and then dry. And I only had, I think it was like five days of wet, and my instructor says, oh, that's not ovulation. [So] I'm like, well, I'm pregnant. And now that I've been in this again, my cycle returned probably six months ago, and I'm fairly confident that I have been ovulating. I see a very clear pattern, but it's not lining up with what she defines as ovulation, so according to my instructor, I still haven't ovulated, and I don't think that's true. (FG3)”

This is consistent with the challenge mentioned by a few participants related to working with instructors who could not teach them to use the Billings Method confidently. In one instance, this involved an instructor who was on practicum, and in another, it involved an instructor who the participant felt was not able to communicate effectively.

“I got lucky with my instructor because she was still training when I took the class with her, so she was still trying to figure [things] out… [So] When I was talking to her about my PCOS, she was very understanding, but… at first, she did not [know] how to approach it. (FG1)”

“I've found that, like [a] communication issue, I don't know, I'm assuming I'm ovulating, but I don't know how to reconcile what I'm experiencing with what my instructor is telling me to expect. (FG3)”

The periods of abstinence required were "hard" and challenging for most users across the groups. However, attitudes towards abstinence varied among participants. A few found these periods of abstinence to be opportunities for practicing self-control and other types of intimacy.

“The one thing that's been a little bit more difficult is the amount of time that we've avoid[ed] intercourse because I am very risk-averse, and of course, as you get more practice, that gets better, and in the beginning, it was a little bit difficult…So, it was hard. We were avoiding for like [about] six months, and I think that was a bit hard for us in our relationship, but it was good because my husband was on-board and he wanted to do it, which was helpful. (FG2)”

“Abstinence is the hard part for both, especially for our husbands. But at the same time, I guess [it] is important because you are in control of your body. Because we are not animals, okay? And that part is important. In my case, it gives me some control; it gives me peace; it gives me trust… When finally, we can [be] together and have sex, [it] is wonderful because it is like when you have a piece of cake, and you are a cake, I like that cake, but I know now I cannot eat it right now, but you wait for that moment, it is more special, you enjoy it more. (FG1)”

### Suggestions for improving the Billings Method delivery

Focus group participants also provided suggestions and recommendations for improving the experience of the Billings Method users. These suggestions, as well as example quotes, are further outlined in Table 5.

**Table 5:** Suggestions

Category		Suggestions	Example Quotes
Teaching & Learning	Expectations of instructors	• Standardize the Billings Method instruction process • Clarify to clients what is included in their fee and that they can ask instructors questions • Have instructors be open to referring clients to others if they are not a good fit • Encourage instructors to continue their education	*I wanted to be sure I can give feedback to help [with] the standardization and that all instructors would be following that process. (FG1)*
	Instructional content & materials	• Better explain/describe the process of observing sensations • Make PowerPoints more visually appealing • Messages to communicate: ○ Potential for environmental factors to affect the cycle ○ Learning the Billings Method is a process ○ Cycles vary • Start teaching girls at a young age; use appropriate language and messaging for younger audiences	*We need to have more grace with ourselves as new users, and our instruction could include that. That your first cycle might not look like this… it's just you're learning how to describe what you're experiencing. (FG2)* *I know there are programs already out there teaching girls Billings at [a] younger age, and I think that is perfect… The earlier, the better cause it's not just about getting pregnant or not getting pregnant; it's about who are you. What is your body telling you?… Using simplified terms like, do you see how nature uses her seasons to change? (FG3)*
	Finding an instructor	• Make lists of instructors available • Include information about instructors on the website • Instructors should communicate about the populations they are comfortable serving (e.g., married v. unmarried, the language of instruction, etc.) • Allow clients to search for instructors based on specialty • Assist with matching clients with instructors (e.g., potential client survey)	*I feel like instructors are similar to therapists, that you need to have a rapport with them, trust, and feel that you can tell them everything… I would like people to know more about me or to have like the little bio of myself on the website … definitely, it would be nice to have an institutionalized way to do it, like here [are] our instructors, here is a little video…here is a bio, what their life looks like… (FG2)*
	Using the Billings Method in the context of relationships	• Offer overview educational materials for partners • Guide couples on other forms of intimacy for periods of abstinence	*It'd be cool if there was just a one-page overview for partners. Like the basics, here's what we're doing, [and] the results we're looking for. (FG4)*
Charting tools		• Better communicate the availability of apps • Improve usability of existing apps • Create apps with features such as: ○ Reminders ○ Mood tracking ○ Partner sharing/communication • Offer easier-to-use charting tools like notebooks or Excel documents • Have example charts available for different situations (e.g., postpartum, Breastfeeding, PCOS, etc.)	*If there was … one standardized booklet that you can use for like five years and keep with you….Cause a loose piece of paper you misplace all the time or the tiny ones you can't even write on it. (FG2)* *I had days where the whole day would go by, and I'd be like… "Did I did I feel wet today?" So… if we're talking about optimizing the app, having a feature that kind of checks in with you. (FG4)* *…Instructors don't even know there is an app to tell their students. (FG1)*
The Billings Method communications	Expanding the Billings Method user base	• Better promote the benefits of the Billings Method (e.g., accuracy, ease, ability to monitor health) to potential users • Expand promotion of The Billings Method outside of the religious sphere; offer non-religious messaging • Have easy-to-understand material on the website	*So, hopefully, Billings could do a better job when they are promoting this Method… "hey, this method is set apart, is different, [if] you don't want to be taking temperatures and you don't want internal investigations, and you still want a 99% accuracy, and you want maybe [to] postpone or prevent or you want to achieve. (FG1)* *In the interest of attracting a wider audience, I find that sometimes there's maybe a little more religious messaging included than necessary…(FG4)*
	Educating healthcare providers on the Billings Method	• Develop a database of healthcare providers who are aware/supportive of the method • Raise awareness/educate healthcare providers about the method	*I feel like just having that awareness out there and make it clear to physicians, practitioners in general; this is NOT what you are thinking, this is NOT the rhythm method, this is not "you count the day 14*^*th"*^*…So, letting them know, hey, this is different. (FG1)* *It would be great if we could have more OBGYNs that know about Billings and are trained or even just have heard about it and read the study before. (FG2)*

**Abbreviations:** *FABM* Fertility awareness-based method, *FG* Focus groups

The Billings Method: Billings Ovulation Method

Many of the suggestions that participants provided were related to teaching and learning in the Billings Method. A few suggestions were made about improving fertility tracking and how the Billings Method is promoted and marketed. Recommendations included: clarifying or standardizing the expectations of instructors; improving instructional content and materials (e.g., important messages to communicate, communicating differently to younger women); streamlining the process of identifying an instructor; providing support for couples who are using the Billings Method in the context of a relationship (e.g., managing periods of abstinence); improving charting tools (e.g., apps); better promoting the benefits of the Billings Method; and offering non-religious messaging to potential users outside of the religious sphere; and raising awareness and knowledge of the Billings Method among educating healthcare providers.

## Discussion

The information obtained from these FGs illuminates the perspectives of women who use FABMs, specifically the Billings Method. Each person’s choice of a family planning method is highly personal. Shared decision-making with healthcare providers is recommended [22]. Many providers prefer to focus on recommending only highly effective methods. However, our findings demonstrate that users who choose the Billings Method have done so for specific reasons, and that once they have chosen this method and continued using it, they remain highly satisfied. Our findings can help providers understand how to counsel patients interested in using the Billings Method and other FABMs in a way that is supportive of their preferences and experiences.

Women in this study chose FABMs -including the Billings Method- not only because of religious beliefs but also because of concerns about side effects of hormonal birth control methods, wanting to use a "natural" method, and a desire to learn more about their bodies. Religious beliefs are one important reason many users choose a natural family planning method, including the Billings Method [23–25]. In our sample, most women were religiously affiliated. This may have been a less frequent theme among a sample of people with few or no religious affiliation. However, in a recent National Survey of Family Growth, users of FABMs did not differ significantly from the other contraceptive users in terms of relationship status, education, parity, health insurance, or religious affiliation [17]. Our findings help shed light on other reasons, besides religious beliefs, for choosing FABMs. Concerns about the side effects of hormonal birth control were mentioned by many in our study and also described by others [23]. In addition, participants brought up the concept of learning a new way to think about themselves through self-awareness, a positive concept previously described by Billings users in Brazil [25].

In the present study, participants expressed that they found the Billings Method easy to understand, simple to use, and affordable, and that they were attracted by the supporting scientific evidence [8, 10, 26–28]. When using the Billings Method for family planning, a few participants in our FGs expressed that achieving pregnancy was "easy," and it has been described that the Billings Method observations can predict successful conception [29]. Some users appear to be interested in using the method because it allows them to use the same method for both planning and avoiding pregnancy. A couple of participants experienced unplanned pregnancies while using the Billings Method. This is not surprising given that FABMs, in general, and the Billings Method, specifically, are characterized by high typical use pregnancy rates. In a recent systematic review by Peragallo Urrutia et al., the pregnancy probabilities with typical use in new users ranged from 10.5 to 33.6 [1]. However, in the same study, authors described effectiveness of 1.1–3.4% if used correctly and consistently [1]. Unlike other methods of family planning, when FABMs are used incorrectly, by definition, intercourse is occurring on a highly fertile day; therefore, typical use pregnancy rates are higher than for methods that do not require as much user input and/or render the user less fertile (e.g., hormonal methods). The participants in our study seemed aware of this characteristic of the Billings Method and were nevertheless satisfied with the method as it fits their other selection criteria. This is consistent with findings from other studies about satisfaction with use of the Billings Method and NFP [30, 31].

Many of our participants also described the importance of their partner's support in using the Billings Method. Including partners in a family planning method, as is necessary for FABM users, may benefit some, especially those highly motivated to use FABMs like the Billings Method. For example, in a qualitative study of Brazilian Billings users, dos Santos et al. (2017) found that the partner's participation fostered dialogue between the couple, helped to identify mucus sensations and made the partner an active participant in family planning [25]. Similarly, in a large multi-country internet-based survey on mostly married couples (89%) using a different type of FABM, symptothermal NFP, Unseld et al. (2017) found that respondents rated partner involvement and commitment in using NFP as "important" or "very important." In addition, 74% of men and 64% of women couples felt that the use of NFP improved their relationship with their partner [32]. Another descriptive survey study found that couples using NFP had strong bonding and increased communication, among other benefits [30]. However, more research is needed to clearly understand how including partners in family planning can be positive for some users and also for which types of users it may not be positive. For example, most of these populations in the previous studies were recruited through NFP/religious organizations, and most were married. Therefore, they may differ from other populations who may not be married or who may not be using FABMs for religious reasons. Furthermore, in relationships where there is no ability to negotiate the timing of intercourse or use other methods, such as barrier methods, FABM cannot be used to avoid pregnancy. For some couples, the partner's involvement in contraception and family planning can become coercive or abusive [33]. Researchers and instructors of the FABMs should be sensitive to this issue as well as positive ways of handling disclosures of intimate partner violence in the setting of research or FABM instruction [34].

Some participants in our study described specific challenges related to using the Billings Method when having PCOS or breastfeeding. PCOS is a hormonal and metabolic disorder characterized by abnormally short or prolonged menstrual periods, excess androgen levels, or polycystic ovaries. In addition, it is associated with alterations in the cervical mucus and infertility [35, 36]. All these factors may contribute to the challenges experienced by Billings users with PCOS. Furthermore, there is minimal data about the effectiveness of the Billings Method for avoiding pregnancy among users with PCOS or long cycles. Similarly, the hormonal changes during breastfeeding may have contributed to challenges using the Billings Method experienced by the women in this study. While a study by Perez et. al (1988) described that women who were taught the Billings Method during the postpartum period had no difficulty recognizing their fertile and infertile days [37], there is limited effectiveness data for users of FABMs, including the Billings Method, in the postpartum period [9].

Finally, participants in our study made interesting and novel suggestions to strengthen the way in which the Billings Method is taught or promoted, such as streamlining the process for identifying an instructor, improving the usability and features of the charting apps, and offering non-religious messaging to potential users. Finding ways to implement these recommendations may improve user experience and increase the appeal of the Billings Method to diverse users. Findings from this study could also inform the teaching and delivery of other FABMs.

### Study limitations

Our study is limited by the following. First, given that we used a convenience sampling strategy, there is likely selection bias. Most of the participants were White, married, and religiously affiliated. Findings might be different in a more diverse group. Additionally, the study was limited to current Billings Method users due to our desire to improve the Billings Method for those presently using it. Therefore, we did not include previous users who were no longer using this method. Future studies could focus on recruiting people who stopped using the Billings Method to learn more about this experience. Likewise, we only enrolled female users of Billings; therefore, we cannot describe the male partner's experience in couples using the Billings Method. Finally, participation was also limited to users who spoke English and were trained by BOMA – USA instructors. Therefore, findings are likely not generalizable to all Billings Method users, including those in other countries or who speak different languages. However, while we included only people proficient in English, several participants spoke more than one language, including several for whom English was not their primary language. In addition, we engaged people with diverse perspectives and backgrounds, including religious and non-religious participants, of different races/ethnicities, with and without children, and with various reproductive health conditions. The participants' demographics are similar to users in other studies of the Billings Method and may be an indicator of populations who currently have access to learning the Billings Method.

## Conclusions

Current users of the Billings Method expressed an overall positive experience with learning and using the method for family planning. However, some challenges in using and learning the Billings Method were identified. Addressing these could offer opportunities to improve how the Billings Method is taught and delivered. Understanding these user perspectives can also enhance healthcare providers' interaction with FABM current or prospective users, including those of the Billings Method.

## Supplementary Information

**Additional file 1.** Focus Group Codebook.

**Additional file 2.** COREQ Checklist.

## Data Availability

The datasets used and/or analyzed during the current study available from the corresponding author on reasonable request.
