# Supplementary material for: Understanding the perspective of women who use the Billings Ovulation Method®: a focus group study
Source: BMC Womens Health. 2023 May 10;23:251. doi: 10.1186/s12905-023-02398-w (PMC10170796; doi:10.1186/s12905-023-02398-w)
Supplement: Supplementary file 1 — Additional file 1. Focus Group Codebook. [file 12905_2023_2398_MOESM1_ESM.docx]

**Additional File 1: Focus Group Codebook**

**Manuscript Title:** Understanding the perspective of women who use the Billings Ovulation Method®: a focus group study.

**Authors:** Montserrat Ayala-Ramirez, MD, MPH; Mary E. Grewe, MPH; Julie Kaiser, PT, DPT; Emily Kennedy, MS; Martha Winn, RN; Rachel Peragallo Urrutia, MD, MSCR.

**Ref: Submission ID** 3b76a1d1-8a3b-4b90-ae77-6a115698df8e

| **Item No.** | **Code Name** | **Definition** |
| --- | --- | --- |
| 1 | Abstinence | Apply this code when participants discuss perceptions or experiences related to practicing abstinence. |
| 2 | Becoming/Being an instructor | Apply this code when participants discuss anything related to their experience being or becoming a BOM instructor. |
| 3 | BOM Image/Communications | Apply this code when participants discuss image and communications from any BOM headquarters, including BOMA-USA, including educational materials they've created, website, etc. |
| 4 | BOM Rules | Apply this code when participants discuss anything related to BOM rules. |
| 5 | Breastfeeding/Postpartum | Apply this code when participants discuss their perceptions of or experience using BOM when breastfeeding or during the postpartum period. |
| 6 | Charting/identifying patterns | Apply this code when participants discuss their perceptions of or experience with charting, including describing sensations, and identifying fertility patterns when using BOM. |
| 7 | Clarity/ease (BOM) | Apply this code when participants discuss the clarity or ease of use of the BOM, including aspects of BOM they found unclear, confusing, or difficult, and aspects of BOM they found clear or easy to understand and implement. |
| 8 | Cost | Apply this code when participants discuss cost related to learning or teaching BOM or other FABM. |
| 9 | Effect on relationships | Apply this code when participants discuss how BOM has affected or not affected their relationships with romantic partner / spouse / significant other. |
| 10 | Effect on understanding body | Apply this code when participants discuss how BOM has affected or not affected their understanding of their body. |
| 11 | Effects/other | Apply this code when participants discuss ways in which BOM has affected or not affected their lives (other than effect on relationships or understanding body). Examples include helping others understand their bodies or relationship with God. |
| 12 | Effectiveness | Apply this code when participants discuss their perceptions or experiences related to the effectiveness (positive or negative) of BOM on achieving or delaying/preventing pregnancy or predicting fertility, or the science behind the method. |
| 13 | Environmental effect on cycle | Apply this code when participants discuss perceptions or experiences related to ways in which environmental stressors (e.g., temperature, chemicals, light, emotional stress) affect their menstrual cycle. |
| 14 | Experiences/perceptions of other FABMs | Apply this code when participants discuss their experiences or perceptions of using other fertility awareness-based methods or FABM broadly (not BOM specifically). |
| 15 | Family/friends perceptions | Apply this code when participants discuss perceptions that family and friends have about BOM or FABM, including when family/friends refer participants to BOM or FABM. |
| 16 | Finding an instructor | Apply this code when participants discuss their experiences or perceptions related to finding a BOM instructor. |
| 17 | Good quote | Apply this code to note particularly salient or powerful quotes. |
| 18 | Having health issues | Apply this code when participants discuss experiencing health issues (e.g., PCOS) in the context of using BOM, FABM, or contraceptives. |
| 19 | Health provider perceptions | Apply this code when participants discuss healthcare providers' perceptions of BOM or FABM, including when healthcare providers refer participants to BOM or FABM. |
| 20 | Identifying sensations | Applying this code when participants discuss experiences or perceptions with identifying sensations when using BOM (e.g., moist, dry). |
| 21 | Learning BOM | Apply this code when participants discuss their experience learning the BOM. |
| 22 | Negative experiences/challenges | Apply this code when participants discuss negative experiences, things they don't like, or challenges related to learning or using BOM. Also include experiences overcoming or addressing challenges. |
| 23 | Partner perception/involvement | Apply this code when participants discuss their partners perceptions of or involvement w/ BOM or FABM. |
| 24 | Perception of contraceptives | Apply this code when participants discuss their perceptions of contraceptives, including hormonal birth control, IUDs, and condoms. |
| 25 | Positive experiences | Apply this code when participants discuss positive experiences, or things they like, related to learning or using BOM. |
| 26 | Reason for participating | Apply this code when participants discuss their reasons for participating in the focus group. |
| 27 | Relationship with instructor | Apply this code when participants discuss their experiences and perceptions related to their interactions with their instructor, including perceived accessibility of their instructor (e.g., available for questions or not). |
| 28 | Selection of method - BOM | Apply this code when participants discuss their process of selecting and/or deciding to use the Billings Ovulation Method (BOM) specifically. Also use this code when participants discuss how they learned about BOM. |
| 29 | Selection of method - FABM | Apply this code when participants discuss their process of selecting and/or deciding to use fertility-awareness based or natural family planning methods generally, or why they chose or didn't choose another FABM method. |
| 30 | Suggestions | Apply this code when participants discuss suggestions for improving the BOM or experiences of BOM users. |
| 31 | Selection of apps | Apply this code when participants discuss their experiences or perceptions of using apps or charting websites as part of practicing BOM. |
| 32 | Using BOM for family planning | Apply this code when participants discuss their experiences or perceptions related to using BOM for family planning (to achieve, prevent, or delay pregnancy). |
| 33 | Using BOM to monitor health/body | Apply this code when participants discuss experiences or perceptions related to using BOM to monitor health or body generally (e.g., to better understand body). |

**BOM =** Billings Ovulation Method

**FABM=** Fertility Awareness Based-Methods

**IUDs =** Intrauterine Devices
